# Seeing Some One

**DOI:** 10.3389/fpsyg.2018.01747

**Published:** 2018-09-13

**Authors:** Wolfgang Prinz

**Affiliations:** Max Planck Institute for Human Cognitive and Brain Sciences, Leipzig, Germany

**Keywords:** action perception, consciousness, selfhood, agency, intentionality, import, export

## Abstract

This paper outlines a light approach to heavy issues of consciousness. The basic claim is that human minds are very much tailored to the requirements of action perception, that is, to what people see when they watch other people acting. I argue that the third-person perspective entailed in action perception offers an easy and more direct access to such enigmatic things as selfhood, intentionality, and agency than the first-person perspective does. In a sense, we get these things for free when we study action perception. I do not claim that the study of action perception can solve (or even dissolve) the enigmata entailed in consciousness. I do claim, however, that it sheds new light on relationships between one’s own mind and other minds.

## From Being to Seeing

Everybody knows that consciousness is a deeply enigmatic thing—a thing that appears to be quite special and unique in an otherwise non-conscious, or conscious-less world. How can we study this enigma? It is fair to say that our intuitions about the nature of consciousness are mainly rooted in our own beliefs, thoughts, and desires, that is, in self-perception from a first-person perspective. We ground, in other words, intuitions about consciousness in our private experience of what it is like and what it takes to be conscious agents. This is what we may call the study of *being someone* (or perhaps, *no-one*, as some would claim). Remarkably, this way of grounding applies not only to folk notions of consciousness but to psychological and philosophical intuitions as well. Many consider it *alternativlos*^1^, to borrow a fashionable German political term. After all, how else could one approach such a private and elusive thing like consciousness?

In fact, I believe that such alternatives do exist and I am going to sketch one here. The approach I am advocating is grounded in social rather than individual experience. More specifically, it is grounded in the way we perceive other people acting from a third-person perspective. Basically, this approach derives intuitions about being someone from intuitions entailed in seeing someone, that is, from what it is like and what it takes to see people acting in their environment. This is what we may call the study of *seeing someone*.

A crucial claim behind the shift from being to seeing is that the study of seeing someone else delivers us very much the same intuitions as the study of being someone ourselves. In a sense, such correspondence is surprising because the information, on which action perception relies, reflects the visual kinematics of body movements but not the mental dynamics of thoughts, beliefs, and intentions. How, then, can being and seeing share common features? Moreover, this shift raises the question of which is the chicken, and which is the, egg. Does the craft of seeing build upon the experience of being someone—or should we even consider the option that seeing comes first and being second? A full account of these issues is provided elsewhere (Prinz, 2012, 2017). In what follows I concentrate on the role of action perception in grounding intuitions about consciousness (and only marginally touch upon the chicken-and-egg problem pertaining to the first- and third-person perspectives).

## Action Perception

In this section I discuss three features that are shared by being and seeing: selfhood, agency, and intentionality. They play a prominent role in both theoretical accounts of first-person conscious experience and descriptive accounts of third-person action performance.

### Selfhood

Many believe that consciousness relies on self-representation and that some kind of implicit self-involvement is key to understanding the conscious nature of mental acts (e.g., Kant, 1781/1787/2003; Brentano, 1874/2014; Metzinger, 1993, 2003, 2009; see also Kriegel and Williford, 2006; Kriegel, 2009; Prinz, 2012, 2017; Miguens et al., 2016). If so, theories of consciousness must explain where self-representations come from, how they get in place, and how they work. At the same time, self-representations play a prominent role in action perception as well. When we see people acting, our perception specifies their actions in terms of three basic elements: agents, movements, and effects. For instance, in grasping a cup for drinking, the agent is perceived to plan, initiate, and carry out the action. Her bodily movements are seen to realize its execution and the resulting effect is understood to instantiate the goal of drinking coffee. Actions, in other words, are perceived as events that emerge from interactions between mental and physical forces entailed in agents, movements, and effects.

To understand an action is therefore tantamount to understanding the interplay between the mental and the physical forces giving rise to it (see Prinz, 2012, Ch. 7; Prinz, 2017). To start with, consider simple physical events. As we know since Michotte’s classical experiments, perception of such events always goes beyond the information given (Michotte, 1946/1963). For instance, when a billiard ball hits another ball and sets it in motion, the perception of such an event is, on the one hand, exclusively grounded in visual information arising from the kinematics of the balls—information that specifies their temporal and spatial coordinates including their derivatives like direction, speed, acceleration, etc. However, on the other hand, perception provides information that goes, in several respects, far beyond the kinematics of the event. For instance, as we know from the classical work of Runeson and Frykholm (1981), perceivers cannot help but see and understand the physical dynamics entailed in the visual kinematics (i.e., the forces and masses involved in generating the kinematic pattern).

Moreover, when it comes to actions, perceivers also see the underlying intentional dynamics^2^. Intentional dynamics goes beyond physical dynamics by also addressing the mental forces that give rise to perceived actions (e.g., the goals and intentions involved in generating the visible kinematic pattern). As we know from Heider’s classical studies, intentional dynamics can also be directly derived from the kinematic pattern entailed in the visual stimulus configuration (Heider, 1944, 1958; Heider and Simmel, 1944). Such mentalizing seems to be automatically (and even inevitably) implicated in event- and action perception. Even when watching nothing but two moving triangles, perceivers cannot help but see them acting as intentional agents (Abell et al., 2000; for demonstration, see G Fan, 2015). They localize the source of actions within what they perceive to be agents—guided by their intentional minds and carried out by their physical bodies. Perceivers are therefore both dualists and interactionists: they distinguish between physical and mental forces but find it natural to see them interacting. Thus, to see someone acting means to perceive an intentional and agentive mental self operating in a physical body and steering its activity.^3^

In sum, action perception implies a mental self which authors the act and generates the mental and physical forces to realize it. In this view, the inferred agentive self takes the role of a control center that mediates between perception and action by taking up information about states of the world and generating actions suited to modulate them. The self is therefore a natural ingredient of action perception: the world of action perceivers is populated with agentive selves surrounding them. These selves are agentive in the sense of authoring (visible) physical actions that are driven by (invisible) mental states and forces.

### Agency

While selfhood pertains to permanent features of agents, agency and intentionality pertain to transient features of their mental and physical acts (traits vs. states; cf. Tamir and Thornton, 2018). The feature of agency addresses the action side of the perception/action interplay. Agency is associated with one of two control modes that can be seen to underlie ongoing action, viz. bottom-up and top-down. In the first case, movements are seen to arise as responses to externally driven states of affairs, whereas in the second they are seen to arise as a means of realizing internally generated intentions. The notion of agency thus reflects the craft of creating intentions and generating actions suited to realize them. A convenient way to think of intentions is to see them as representations of desired states of affairs, and a convenient way to think of actions is to see them as physical means for coming close to these states.

Agency is often regarded as a hallmark of the human condition. Still, the notion of agency poses two basic problems, an easy and a not-so-easy one. The easy problem concerns the transition from (mental) intentions to (physical) actions. At least in principle, this problem can be solved since intentions and actions can both be expressed in representational terms. The not-so-easy problem pertains to the purported generation of intentions from within. It can be read strongly or weakly. The strong reading invokes free will, that is, some kind of undetermined creation of intentions *ex nihilo*. This is, of course, a reading that scientists and many philosophers want to avoid. The weak reading claims that intentions are created by some kind of sub-intentional machinery which explains why they look as if they were created *ex nihilo*.

In any case, agency is a feature that observers cannot help but *see* when they watch other people acting. As indicated above, intentional dynamics are often directly specified by the kinematic pattern entailed in the stimulus configuration. For instance, when we see someone lifting a box and putting it on a table, we cannot help but see her lifting the box for the sake of putting it on the table. Likewise, when we watch nothing but two triangles moving relative to each other in particular ways we cannot help but see one chasing the other or trying to escape.

Agency is, of course, closely related to selfhood. However strong or weak the reading may be, the source of the intention is always attributed to the authoring agent. Again, this is true of both my own intentions (which I know from within) and someone else’s intentions (which I can see from without).

### Intentionality

The notion of intentionality addresses the perceptual side of the perception/action interplay. How is it possible for states in our mind to refer to things in the world, say, a tree in a meadow or a sore on my arm? There are actually two questions here that are both considered deeply enigmatic, a general and a specific one (Brunswik, 1952; Dretske, 1981; Grice, 1991; Prinz, 1992). The general question pertains to intentionality proper, that is, the issue of representational reference. How is it possible for one thing to refer to an entirely different thing in the sense of having it as content? The specific question refers to distal reference. How is it possible for mental things to skip over proximal things and refer to distal things? For instance, why do we see the tree *in the meadow* or the sun *in the sky*? Why do we see these things out there and why don’t we see, for example, the retinal activation patterns or the brain states involved in generating these percepts? This seems to pose a paradox: How can representations skip over proximal links in the chain of events leading to them and single out a particular distal link for representational reference?

As long as we think of intentionality and distal reference from the first-person perspective of being someone, there is no obvious way to resolve the paradox. However, the paradox goes away when we adopt the third-person perspective of seeing someone acting, and interacting, with the world. From this perspective, intentionality and distal reference appear to be natural ingredients, rather than paradoxical features, of conscious experience. The basic idea is illustrated in Graziano’s story about Bill who looks at a cup and Abel who watches Bill doing so (Graziano, 2013, p. 86). In this scenario, the observer (Abel) construes the other (Bill) as having states in his mind that address things in their shared environment (the cup). In other words, by virtue of seeing Bill acting in the scene, Abel understands Bill’s percepts, concerning the cup, as private states in Bill’s mind that refer to public things in their shared environment. Observers thus construe mental states of others as private states in the others’ minds that exhibit representational reference to public things in the world. Their proper function is to refer to things out there, with no links in-between mediating that referential relationship. Once again, things that are difficult to understand from a first-person perspective appear to be natural and obvious from a third-person perspective.

### How Come?

If it is true that action perception provides us with such nice things like selfhood, agency, and intentionality, we may wonder how such a miracle can happen. Aren’t we shifting the enigma of consciousness from first-person perception to the domain of third-person perception? In a sense, we are, but there is a critical difference since much of the enigmatic aura gets lost in the move. This is because we know, at least in principle, how action perception works. We can understand that selfhood, agency, and intentionality are ordinary categories of action perception—categories whose functioning is by no means mysterious.

Think again of what we have learned from classical experiments on event perception (Michotte, 1946/1963; Shipley, 2008): Perceivers see and understand the non-visual dynamics entailed in the visual kinematics (i.e., the forces and masses involved in generating the spatio-temporal pattern). Such perceptual enrichment seems to come for free—effortlessly, automatically, and even unavoidably.

Remarkably, such automatic perceptual enrichment applies not only to physical events but also (or even more so) to actions (Johansson, 1973; Runeson and Frykholm, 1981). To understand an action is tantamount to understanding the interaction between the underlying mental and physical dynamics. Physical dynamics pertains to the forces that underlie and generate the kinematics entailed in the stimulus configuration. Intentional dynamics goes beyond physical dynamics by also addressing the mental forces giving rise to the actions we perceive. As said above, physical and intentional dynamics both come for free in the act of perceiving since they are both directly specified by the kinematic pattern entailed in the stimulus configuration.

Of course, there is much more to be said about the workings of action perception. For instance, one point that needs careful consideration pertains to the danger of circularity of the argument. Doesn’t perceiving selfhood and intentionality in others already require, and presuppose, conscious awareness in the perceiver? The answer depends on what we mean by perceiving, or seeing. If we use these terms to address conscious personal acts, the explanation is certainly circular. However, this is not the case if we claim that perceiving, or seeing initially rely on automatic sub-personal operations for capturing the meaning of events and actions without awareness. In any case, in order to explain how action perception can lay the ground for conscious awareness, we need to resort to non-conscious ways of perceiving.

## From Seeing to Being

To conclude, let me briefly discuss two questions concerning broader implications. The first comes back to relationships between being and seeing that we have already touched upon. The second addresses a novel issue pertaining to relationships between models and reality.

### Export vs. Import

First, what can we conclude from the discovery of key features of first-persons conscious experience in the perception of third-person action performance? The most obvious and most general conclusion seems to be that the divide between first- and third-person perspectives must be not as deep and categorical as is often thought. The fact that the perspective of being someone is fundamentally different from the perspective of seeing someone does not necessarily imply that the things that the two perspectives deliver must be fundamentally different as well. At least from a commonsense perspective, it is not overly surprising that we understand others as we understand ourselves and vice versa.

Still, how can such correspondence emerge? As discussed elsewhere we may think of two hypothetical pathways, export and import (Prinz, 2017). Export theorists claim that we model others after ourselves in that we find things like selfhood, agency, and intentionality in others after and because we have encountered them in ourselves. By contrast, import theorists believe that we model ourselves after others such that we build up selfhood, agency, and intentionality in ourselves after and because we have encountered them in others.

The export view, which moves *from being to seeing*, has been mainstream for centuries. It builds on the intuition that individuals can only be at home in their own minds, but not in foreign minds. If so, the sole way to understand others is to export one’s own mentality to them, that is, to impute one’s own mental resources on others. By contrast, the import view, which moves *from seeing to being*, has played only a marginal role until now. It builds on the intuition that individuals may first detect and understand subjectivity and selfhood in others, before they then begin to import this understanding for developing their own mentality. The list of supporters of an import view includes philosophers like Smith (1759/1976), Hegel (1807/1977), Sellars (1963) and Carruthers (2009, 2011) and social and developmental scientists like Vygotsky (1925/1979), Mead (1934), and Taylor (1989). These authors share the idea that individuals may first detect and understand subjectivity and selfhood in others before they then import them for building up their own mentality.

As argued elsewhere, I believe that the power of import theory has been notoriously underestimated and that we have good reasons to take a fresh look at it (Prinz, 2012, 2017). Such a fresh look must—among many other things—solve the puzzle of how it is at all possible that representations derived from seeing someone else can lay the ground for being someone oneself. How is it possible for representations to create reality?

### Fact vs. Fiction

At first glance, this sounds fairly mysterious: Aren’t we just creating a new enigma? I don’t think so. On a closer look, the mystery goes away when we put this claim in the broader context of a theory of social facts and underlying ontologies.

While export theorists believe in a primary, natural self, which we first perceive in our own minds and then export to other minds, there is no such thing as a primary self in import theory. What import theory offers instead are self-representations that are derived from other minds and then imported and implemented in one’s own mind. However, representations of selves are not the same things as real selves. Could it therefore be, that an imported self is nothing but a beautiful chimera—a collectively shared illusion that has nothing to do with the true workings of the machinery of the mind? Could it be that we all share the illusion of being someone, when in fact we are no one (Metzinger, 2003)? Are we talking about fictions rather than facts?

As I said, I contend that this concern is unfounded. Basically, there are two arguments that make it so—a general argument which applies to all social artifacts and a specific one that relates to self-referential artifacts like selfhood and subjectivity. The general argument pertains to the ontological status of social facts and artifacts. In the realm of social artifacts, which are created by social exchange, reality is always created by representations. For example, being something or someone may arise from seeing or being seen as, something or someone, respectively. Social artifacts are, in Ian Hacking’s terminology, interactive kinds—categories that when used act back on the entities that they categorize (Hacking, 1999). These entities become real and efficacious *in virtue* of categorizations pertaining to them and *in virtue* of the values and beliefs entailed in these categorizations. Bank notes are valuable because they are categorized as such and therefore considered valuable. Judges preside over courts because we consider them competent and qualified to do this. Popes, presidents, and prime ministers *are* popes, presidents, and prime ministers because we acknowledge and recognize them to be this. In exactly the same way, selves are real and efficacious because we and they themselves see and believe them to be. Like any other social institution, the mental self is therefore in no way fictitious or illusory but instead absolutely real—a real artifact, as Kusch states (Kusch, 1997).

Added to this—and this is the special argument—is the fact that subjects are auto-artifacts and therefore systems that observe themselves and develop representations of their own activities. As we know from Luhmann’s systems theory, self-observation can become operative in such auto-referential systems (Luhmann, 1984/1995). That means that representations which systems develop of their own activity, may acquire the power to control and modulate this activity. To make this abstract principle work, we need, of course, to spell out the concrete details of the mechanisms through which such self-representations become real and efficacious (Prinz, 2012, 2017).

Fact or fiction? Are mental selves real after all? People have a self in the same sense as they have, for example, money, courts of law, and governments. Money, courts of law, and governments are social institutions which they create and recognize. The same applies to mental selves. Once implemented, they are in no way fictitious or even illusory. Rather, they are real facts which determine and constrain our range of action, in the same way as do the facts of the natural surroundings in which we live.

## Author Contributions

The author confirms being the sole contributor of this work and approved it for publication.

## Conflict of Interest Statement

The author declares that the research was conducted in the absence of any commercial or financial relationships that could be construed as a potential conflict of interest.

## Footnotes

1 Which means that this way is void of alternatives—the sole way one can go.

2 Note that perceiving an action as being generated by certain intentional dynamics does not mean that this perception is infallible.

3 Again, perceiving someone as the author of an action does not mean that this perception is infallible. Accordingly, as concerns the perceived agent, I don’t mean to endorse any claims pertaining to relationships between agency and authorship.
